# DNA Methylation Patterns of a Satellite Non-coding Sequence – *FA-SAT* in Cancer Cells: Its Expression Cannot Be Explained Solely by DNA Methylation

**DOI:** 10.3389/fgene.2019.00101

**Published:** 2019-02-12

**Authors:** Daniela Ferreira, Ana Escudeiro, Filomena Adega, Raquel Chaves

**Affiliations:** ^1^Laboratory of Cytogenomics and Animal Genomics, Department of Genetics and Biotechnology, University of Trás-os-Montes and Alto Douro, Vila Real, Portugal; ^2^BioISI – Biosystems & Integrative Sciences Institute, Faculty of Sciences, University of Lisboa, Lisbon, Portugal

**Keywords:** *FA-SAT* DNA, *FA-SAT* non-coding RNA, DNA methylation, satellite DNA, cancer

## Abstract

Satellite ncRNAs are emerging as key players in cell and cancer pathways. Cancer-linked satellite DNA hypomethylation seems to be responsible for the overexpression of satellite non-coding DNAs in several tumors. *FA-SAT* is the major satellite DNA of *Felis catus* and recently, its presence and transcription was described across Bilateria genomes. This satellite DNA is GC-rich and includes a CpG island, what is suggestive of transcription regulation via DNA methylation. In this work, it was studied for the first time the *FA-SAT* methylation profile in cat primary cells, in four passages of the cat tumor cell line FkMTp and in eight feline mammary tumors and the respective disease-free tissues. Contrary to what was expected, we found that in most of the tumor samples analyzed, *FA-SAT* DNA was not hypomethylated. Furthermore, in these samples the transcription of *FA-SAT* does not correlate with the methylation status. The use of a global demethylating agent, 5-Azacytidine, in cat primary cells caused an increase in the *FA-SAT* non-coding RNA levels. However, global demethylation in the tumor FkMTp cells only resulted in the increased levels of the *FA-SAT* small RNA fraction. Our data suggests that DNA methylation of *FA-SAT* is involved in the regulation of this satellite DNA, however, other mechanisms are certainly contributing to the transcriptional status of the sequence, specifically in cancer.

## Introduction

In recent years, satellite non-coding RNAs (satncRNAs) have emerged as cancer key players (Ferreira et al., 2015). Different studies have related the overexpression of these sequences with their hypomethylation status in cancer (Ehrlich, 2009; Saksouk et al., 2015), being its aberrant expression observed in a variety of cancers (Ting et al., 2011) and associated with genomic instability (Bouzinba-Segard et al., 2006; Valgardsdottir et al., 2008). However, the works of satncRNAs on cancer cells are still scarce to really understand the putative mechanisms that control the transcription of these sequences and therefore their involvement in the cancer process.

*FA-SAT* was described as the major satellite DNA (satDNA) family of *Felis catus* (FCA, the domestic cat) (Fanning, 1987), displaying a primary location at the telomeres and a secondary location at the centromeres of some chromosomes (Santos et al., 2004). The amplification of this sequence in a fibrosarcoma was suggested to be associated with the mitotic instability found in that tumor cells (Santos et al., 2006). Additionally, and recently, Chaves et al. (2017) also described the presence of this satDNA in an interspersed fashion in all the cat chromosomes using an *in silico* approach on the cat whole genome sequencing data (felis_catus_8.0; GenBank, assembly accession: GCA_000181335.3). It was also demonstrated that this sequence is present, highly conserved and transcribed in several Bilateria species, what anticipates an important function for its transcripts (Chaves et al., 2017). Furthermore, *FA-SAT* is a GC-rich satDNA (Fanning, 1987; Pontius and O’Brien, 2009; Chaves et al., 2017) and a CpG island was already identified in its monomeric unit, being its DNA methylation status similar in all the Bilateria species analyzed by Chaves et al. (2017), suggesting that the expression of *FA-SAT* can be regulated by DNA methylation events. In fact, some studies already proved that satDNA sequences are regulated by DNA methylation and are frequently hypomethylated and as a consequence, overexpressed in cancer (Ehrlich, 2009; Ferreira et al., 2015; Saksouk et al., 2015).

In this work, the study of the DNA methylation profile of *FA-SAT*, its expression and copy number variation in cancer and non-cancer cells is presented for the first time. In order to disclose if *FA-SAT* is regulated by DNA methylation mechanisms, we designed an approach that includes the simultaneous analysis of different cellular models: a cat primary cell culture (non-tumor cells, FCAn); four passages of the feline mammary tumor (FMT) cell line, FkMTp (distributed over time) (Borges et al., 2016); and eight FMT and the respective disease-free tissues (DFT). The different passages of the FMT cell line allowed us to perceive the behavior of these cells over time with respect to the acquired cancer-driver mutations. On the other hand, inclusion of spontaneous malignant cat tumors was due to the fact that, as described in the literature, the tumor microenvironment can influence the methylation status of the genome and this is absent in the *in vitro* cultured cells (Ting et al., 2011). As already mentioned, *FA-SAT* DNA amplification was described in a fibrosarcoma (Santos et al., 2006). Additionally, satDNA sequences are known to be physical hotspots for karyotype rearrangements in cancer (Jackson et al., 2004; Murphy et al., 2005; Lopez-Flores and Garrido-Ramos, 2012). Thus, we first analyzed the main physical location of *FA-SAT* DNA in the different passages of the cancer cell line FkMTp by DNA-FISH. The *FA-SAT* Copy Number Variation and long RNA levels were also accessed in all the samples. Furthermore, we also quantified the *FA-SAT* ncRNAs in the small RNA fraction (<200 bp), since it was described that the same satDNA can originate small and long satellite transcripts (Bouzinba-Segard et al., 2006; Lu and Gilbert, 2007). The data were integrated with the DNA methylation status of all the analyzed samples and a global demethylation assay was performed on the cancer cell line, allowing to better understand the influence of the DNA methylation mechanism on the regulation of *FA-SAT* in cancer cells.

## Materials and Methods

### Cell Culture and Treatments

*Felis catus* primary cell culture (FCAn) and the different passages of FkMTp cell line were grown in DMEM supplemented with 13% AmnioMax C-100 Basal Medium, 2% AminoMax C-100 supplement, 10% FBS, 100 U/mL/100 μg/mL of Penicillin/Streptomycin antibiotic mixture and 200 mM L-Glutamine (all from Gibco, Thermo Fisher Scientific). FCA primary cell culture was established by our group and was derived from a disease-free mammary biopsy of a female *Felis catus* individual. FkMTp mammary tumor cell line was also established by our group from a mammary tumor biopsy of a female *Felis catus* individual and is already properly characterized (Borges et al., 2016). In this work, the passages analyzed were p7, 21, 70, and 112. Each of these passages was cultured from no more than four passages. For global genome demethylation, complete medium was supplemented with 3 μM of 5-Azacytidine (5-AZA) (Sigma Aldrich) for 72 h. Every 24 h, the 5-AZA medium was replaced. Additionally, all the cells were grown without 5-AZA as experiment controls. For its analysis a sample of the cells was collected for DNA and RNA isolation.

### Mammary Tissue Collection

This study included eight spontaneous mammary malignant tumors of different grades (I to III) from female cats and the respective disease-free tissues (DFT) received from different veterinary hospitals or private practices for diagnosis and treatment. The owners gave consent for the collection of disease-free tissues and tumor samples, accepting that these might be used for research purposes. All the samples were obtained in accordance with the EU Directive 2010/63/EU. All the tumors were histologically classified according to the World Health Organization (WHO) criteria of dog and cat mammary neoplasms. During the chirurgical procedure, the fresh tumors and the normal tissues were immediately preserved in an RNA stabilization solution (RNA Later Tissue Collection, Ambion) and frozen at -80°C to prevent RNA degradation by RNases.

### DNA-FISH

Physical mapping of *FA-SAT* onto chromosomes was made by FISH applying routine procedures (Heslop-Harrison and Schwarzacher, 2011). PCR was used to label a *FA-SAT* cloned sequence with digoxigenin-11-dUTP (Roche Biochemical reagents, Sigma-Aldrich). The most stringent post-hybridization wash was carried out at 50% formamide/2 × SSC at 42°C. *FA-SAT* probes’ hybridization was detected with antidigoxigenin-5′-TAMRA (Roche Biochemical reagents, Sigma-Aldrich) and the preparations were mounted and counterstained with Vectashield mounting medium containing 4′-6-diamidino-2-phenylindole (DAPI) (Vector Laboratories).

### Isolation of DNA and RNA

Genomic DNA isolation was performed using the Quick-Gene DNA Tissue Kit S (Fujifilm Life Science), following the manufacturer’s instructions. Total and small RNA fractions were isolated using the mirVana Isolation Kit (Ambion, Thermo Fisher Scientific) following the manufacturer’s recommendations. Total RNA was purified using the TURBO DNA-free^TM^ Kit (Ambion, Thermo Fisher Scientific). The DNA and RNA quantification was performed using NanoDrop 1000 (Thermo Fisher Scientific).

### Bisulfite Conversion and Sequencing

The DNA methylation status of the *FA-SAT* DNA sequences was analyzed by sodium bisulfite conversion and sequencing. The sodium bisulfite conversion was carried out using the Cells-to-CpG^TM^ Bisulfite Conversion Kit (Applied Biosystems, Thermo Fisher Scientific), following the manufacturer’s instructions. The converted DNA was then used to amplify *FA-SAT* by PCR using bisulfite sequencing PCR (BSP) primers (Supplementary Table 1) with a specific amplification that includes the CpG-rich region. The PCR conditions included an initial denaturation at 94°C during 3 min and 30 cycles of denaturation at 94°C for 1 min, followed by annealing at 57°C for 45 s and extension at 72°C for 45 s and then a final extension at 72°C for 10 min. The amplicons of each sample were purified from the agarose gel using the QIAquick PCR purification kit (Qiagen), cloned into the vector Puc19SmaI (Fermentas, Thermo Fisher Scientific) and sequenced (minimum of 10 different clones). The fragments were then analyzed by MethylViewer software (Pardo et al., 2011). The DNA methylation percentage results are based in the analysis of all the different clones, resulting in a more accurate approach. A cut-off ≥ 20% was considered as biologically significant.

### *FA-SAT* DNA Copy Number Absolute Quantification

For *FA-SAT* copy number absolute quantification (primers in Supplementary Table 1) the standard curve method was used as described in Chaves et al. (2017). The MeltDoctor HRM Master Mix, which uses the SYTO9 dye (Applied Biosystems, Thermo Fisher Scientific) was used for the reactions following the manufacturer’s recommendations. StepOne real-time PCR system (Applied Biosystems, Thermo Fisher Scientific) was the equipment used and the program was: initial denaturation at 95°C (10 min), and then to 40 cycles at 95°C for 15 s followed by 59°C for 45 s and 72°C for 1 min. Subsequently, a melt curve was performed to evaluate the primers’ specificity. All reactions were performed in triplicate and negative controls (without DNA) were also included in the plate. StepOne software (version 2.2.2, Applied Biosystems, Thermo Fisher Scientific) allowed to create the standard curve (Supplementary Table 2) and to perform data analysis. The absolute quantification was transformed in fold-changes using the standard curve equation and always in comparison with a control sample. A cut-off ≥ 2 times was considered as biologically significant.

### Real-Time RT-qPCR

For *FA-SAT* RNA quantification (primers in Supplementary Table 1) the standard curve method was used as described in Chaves et al. (2017). Standard curve parameters are referred in Supplementary Table 2. Verso 1-Step RT-qPCR kit, SYBR Green, ROX (Thermo Scientific) was used for the expression quantification, following the manufacturer’s instructions. The reactions were carried out in a 48-well optical plate (StepOne real-time PCR system, Applied Biosystems, Thermo Fisher Scientific) at 50°C for 15 min and 95°C for 15 min, followed by 40 cycles of 95°C for 15 s, 59°C for 45 s and 72°C for 1 min. Subsequently, a melt curve was generated to evaluate the primers specificity. All reactions were performed in triplicate, and negative controls (without RNA) were also included in the plate. The data were analyzed using the same parameters and the StepOne software (version 2.2.2, Applied Biosystems, Thermo Fisher Scientific). A cut-off ≥ 2 times was considered as biologically significant.

### Statistics

All data from Copy Number Variation and Real time RT-qPCR analysis are based on three replicates how good practice requires and are presented as mean ± standard deviation (*SD*). The software used to analyze these data and perform the graphics was GraphPad Prism 6. Statistical significance was determined using two-tailed Student’s *t*-test for the comparison between two independent samples and analysis of variance (ANOVA) tests when more than two groups were under analysis. ns *p* > 0.05, ^∗^*p* ≤ 0.05, ^∗∗^*p* ≤ 0.01, ^∗∗∗^*p* ≤ 0.001, ^∗∗∗∗^*p* ≤ 0.0001.

## Results

### *FA-SAT* DNA Amplification Is Not Associated With Its Overexpression in FkMTp

**FIGURE 1:**
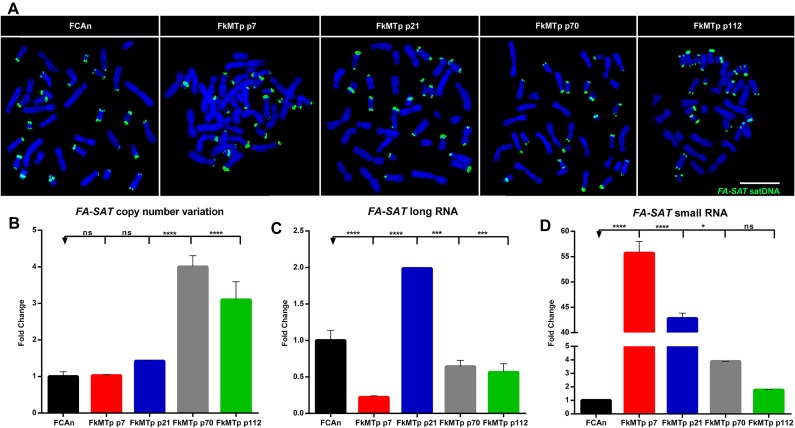
*FA-SAT* DNA and RNA profiles across the FkMTp cell line passages. **(A)** Physical mapping of *FA-SAT* DNA by *in situ* hybridization (green) onto chromosomes (blue) from FCAn (non-tumor FCA primary cells) and from the different passages of FkMTp (p7, p21, p70, and p112). Scale bar represents 10 μm. **(B)** *FA-SAT* copy number fold change in the different passages of FkMTp considering FCAn as the reference genome. **(C,D)** Relative quantification of *FA-SAT* long **(C)** and small **(D)** ncRNA in the different passages of FkMTp, using FCAn as the reference. Values are mean ± SD of three replicates. ns, non-significant ^∗^*p ≤* 0.05, ^∗∗∗^*p ≤* 0.001, ^∗∗∗∗^*p ≤* 0.0001 as determined by one-way ANOVA.

To dissect the regulation mechanisms of a satDNA sequence, it is essential to characterize its DNA and ncRNA profile. This encompasses the analysis of the *FA-SAT* DNA copy number and chromosome location (in FCAn and FkMTp cells), and the quantification of *FA-SAT* ncRNA levels. The analysis of the *FA-SAT* DNA location in cat chromosomes from the FCAn and FkMTp cells by FISH (Figure 1A) showed that there is no evident alteration on the *FA-SAT* main location neither sequence amplification in the different passages of FkMTp cells. Although this cell line exhibits a highly rearranged composite karyotype (Borges et al., 2016), it seems that the physical location of the *FA-SAT* DNA arrays was not changed in comparison to the normal/standard situation (i.e., at the telomeres and/or chromosomes’ centromeres). However, due to the FISH resolution limitations, it is not possible to completely establish if *FA-SAT* DNA was affected by the karyotype reshuffling events occurred during the tumor progression.

In order to accurately analyze the copy number variation of *FA-SAT* DNA in FkMTp compared to primary FCA cells (FCAn), a qPCR quantification was performed in real-time. In Figure 1B and Supplementary Table 3 is possible to observe that *FA-SAT* DNA is amplified in p70 and p112, specifically 4.0 and 3.1 times. This increase may either reflect amplifications of *FA-SAT* globally throughout the genome or instead, amplifications of sequences at its preferred chromosome locations (i.e., telomeric or centromeric), undetectable by FISH, as referred above.

The *FA-SAT* ncRNA levels were analyzed in all FkMTp passages and in FCAn (used as reference). As can be observed (Figure 1C and Supplementary Table 3), the passages that present *FA-SAT* DNA amplification (i.e., FkMTp p70 and p112) do not seem to be overexpressing *FA-SAT*. In fact, only p21 showed to have a high level of *FA-SAT* ncRNA. We also quantified the *FA-SAT* ncRNAs in the small RNA fraction (<200 bp), being possible to detect an increased amount of *FA-SAT* small ncRNA in p7 (55.7 times) and in p21 (42.8 times) in Figure 1D (Supplementary Table 3). Furthermore, when the levels of *FA-SAT* transcripts in the total and small fractions are compared inside each sample, the amount of *FA-SAT* small RNA is higher than the *FA-SAT* long transcripts in the initial passages of the tumor cells (p7 and p21) and it is decreased in FCAn and in FkMTp p112 (Supplementary Figure 1). There is no evidence that the *FA-SAT* overexpression (long and/or small transcripts) observed is related with the amplification of *FA-SAT* DNA (i.e., p70 and p112). These observations suggest that other regulation mechanism(s) may be involved in the transcription of this sequence, as DNA methylation.

### *FA-SAT* Does Not Appear to Be Simply Regulated by DNA Methylation Events in FkMTp Cells

**FIGURE 2:**
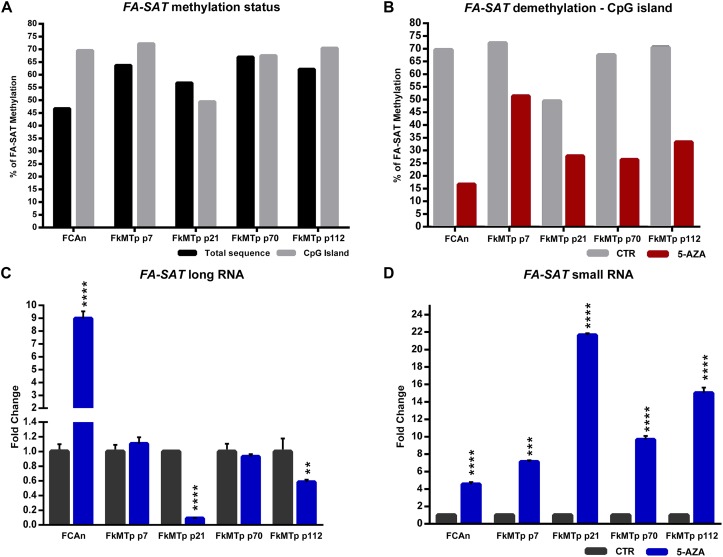
*FA-SAT* is regulated by DNA methylation. **(A)** Graphical representation of the *FA-SAT* methylation percent values regarding the total 15 CpG sites analyzed (Total) and the 8 CpG island sites analyzed by bisulfite sequencing (Supplementary Figure 2a) of FCAn and of the different passages of FkMTp. **(B)** Graphical representation of the *FA-SAT* methylation percent values in the CpG island observed by bisulfite sequencing (Supplementary Figure 2a, analysis of the 15 CpG sites in Supplementary Figure 2c) of FCAn and the different passages of FkMTp in the control (CTR) and in the global demethylation of the genome (5-AZA). **(C,D)** Relative quantification of *FA-SAT* long **(C)** and small **(D)** RNA in FCAn and in the different passages of FkMTp in the azacytidine treatment using the respective control (without AZA) as reference. Values are mean ± SD of three replicates. ^∗∗^*p ≤* 0.01, ^∗∗∗^*p ≤* 0.001, ^∗∗∗∗^*p ≤* 0.0001 as determined by Student’s *T*-test.

The DNA methylation status analysis of *FA-SAT* in FCAn and in the FkMTp passages was performed by bisulfite sequencing. In Figure 2A (and Supplementary Figure 2a) is shown the analysis of the total CpG sequence sites (15 CpG sites) and specifically the 8 CpG island sites (the design of the *FA-SAT* CpG island can be checked Supplementary Figure 2b). In a general analysis, the CpG island showed similar or higher methylation percentages than the total CpG sequence analyzed and most of the cell line passages demonstrated to be methylated (values higher than 50%, ranging from 67.5 to 72.2%). Exception goes to FkMTp p21 that presented the lowest percentage of methylation (49% in the CpG island). In fact, this is the only FkMTp passage that shows overexpression in the *FA-SAT* long RNA fraction (Figure 1C). These data suggest that DNA methylation is, at least, one of the mechanisms responsible for the *FA-SAT* transcription regulation. In order to validate this hypothesis, we performed an assay using 5-Azacytidine (5-AZA), a global genome demethylation agent. In fact, the analysis of the methylation status before and after the 5-AZA treatment, showed that this agent was successful in demethylating the *FA-SAT* DNA sequences (Figure 2B and Supplementary Figures 2a,c). If DNA methylation is responsible for the regulation of this satDNA sequence, its demethylation should unleash the *FA-SAT* transcription. Indeed, in the FCA primary cells, the demethylation of *FA-SAT* sequence caused overexpression of *FA-SAT* of both long and small transcripts (Figure 2C,D and Supplementary Table 4). However, this was not observed in FkMTp passages (Figure 2C,D and Supplementary Table 4), at least for the *FA-SAT* long transcripts, whose levels didn’t increased when *FA-SAT* DNA was demethylated. Moreover, the FkMTp p21 cells, which showed a *FA-SAT* overexpression in untreated cells (without 5-AZA treatment) (Figure 1C) exhibited the lowest levels of *FA-SAT* long ncRNAs when the sequence was demethylated (Figure 2C). Curiously, all the FkMTp passages analyzed revealed *FA-SAT* overexpression of the small RNA fraction (Figure 2D), with p21 showing the highest level. The different behavior of the cancer cell line and the FCA primary cells certainly reflects mutations acquired by the cell line and that must have affected cellular pathways, which in turn caused the overexpression of the *FA-SAT* ncRNA in the small RNA fraction. Thus, our data suggest that although DNA methylation is regulating *FA-SAT* transcription in primary cells (FCAn), another mechanism must be involved in the regulation of this satDNA in cancer cells; alternatively, in the cancer cell line, the DNA methylation events in the *FA-SAT* sequences are dysregulated due to mutations acquired throughout the “*in vitro* process of tumorigenesis.”

### The *FA-SAT* DNA Hypomethylation Is Not the Single Mechanism Responsible for Its Upregulation in Feline Mammary Tumors

**FIGURE 3:**
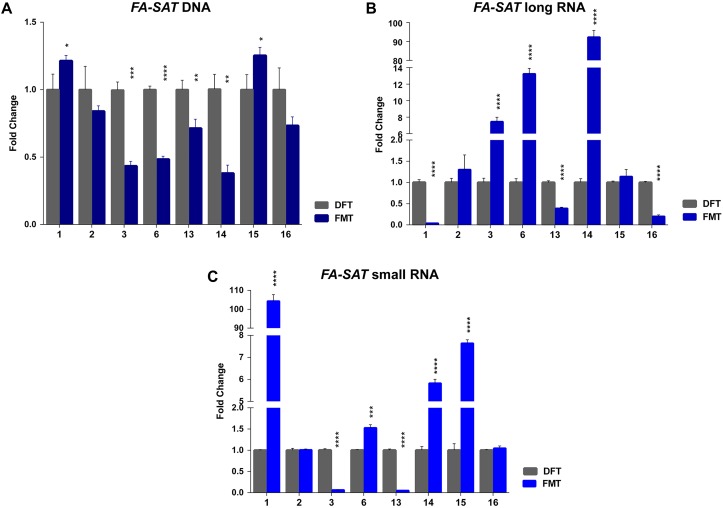
*FA-SAT* DNA and RNA profiling in feline mammary tumors. **(A–C)** Fold change of *FA-SAT* DNA **(A)** and *FA-SAT* long **(B)** and small RNA **(C)** in feline mammary tumors (FMT) by real-time qPCR (DNA) and RT-qPCR (RNA) using a disease-free tissue (DFT) sample of the same individual as reference. Values are mean ± SD of three replicates. ^∗∗∗^*p* ≤ 0.001, ^∗∗∗∗^*p* ≤ 0.0001 as determined by Student’s *t*-test.

In this study, a collection of eight spontaneous feline mammary malignant tumors (FMT) and the respective disease-free tissue (DFT) samples were also included. The analysis of the *FA-SAT* DNA copy number revealed its loss or maintenance in the tumor samples (Figure 3A and Supplementary Table 5). These observations contrast with results of DNA amplification observed in other satDNAs (Savelyeva et al., 1994; Bersani et al., 2015), in the *FA-SAT* DNA in a fibrosarcoma (Santos et al., 2006), and in some passages of the tumor cell line FkMTp (Figure 1B and Supplementary Table 5).

**FIGURE 4:**
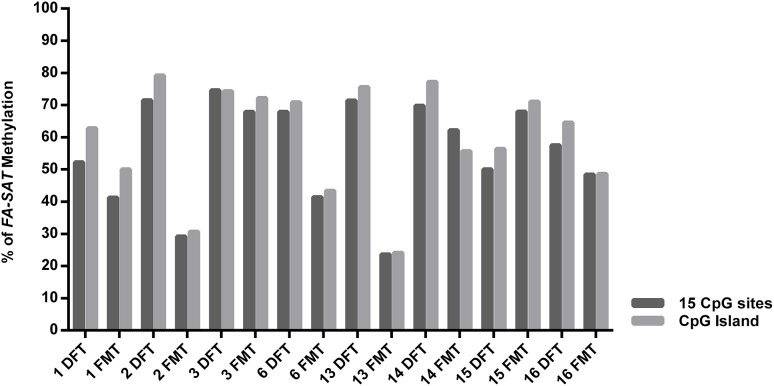
*FA-SAT* methylation status in feline mammary tumors. Graphical representation of the *FA-SAT* methylation percent values regarding the total 15 CpG sites analyzed (Total) and the 8 CpG island sites analyzed by bisulfite sequencing (Supplementary Figure 3) of each FMT and the respective DFT.

The quantification of *FA-SAT* long and small transcripts revealed the occurrence of tumor samples presenting either increase, maintenance or decrease of the levels of these ncRNAs (Figure 3B,C and Supplementary Table 5), representing an adequate set of samples for this study. In general, and similarly to what was observed for the FkMTp passages, in the tumor tissues the *FA-SAT* DNA and the respective levels of ncRNAs revealed no association, most likely being other mechanisms involved in the regulation of their transcription. Consequently, the *FA-SAT* DNA methylation status was analyzed in the tumor and disease-free tissue samples in order to relate these data with the *FA-SAT* DNA and RNA profiles (Figure 4, Table 1, and Supplementary Figure 3). In fact, this sequence is hypomethylated in half of the tumors and maintains the DNA methylation status (compared to the DFT counterpart) in the remaining ones. The CpG island methylation percentage ranges from 56.4–76.4% in DFT samples and 24.1–72.2% in FMT. Table 1 integrates all the data obtained for the *FA-SAT* DNA and RNA (long and small) status (maintenance, increase or decrease when compared with its DFT control, cut off ≥ 2) and the *FA-SAT* DNA methylation status (cut off ≥ 20%). In most of the tumor samples analyzed, there is not a direct correlation between the *FA-SAT* methylation status and its ncRNA levels (Table 1). Only two samples (designated as 6 and 14), present a clear association. Similarly to what was observed for the FkMTp cancer cell line (previous result section), these data suggest that other mechanisms than DNA methylation must be involved in the transcriptional regulation of *FA-SAT* in FMT tissues or alternatively, the DNA methylation events in the *FA-SAT* sequences are dysregulated due to mutations acquired throughout the “*in vivo* process of tumorigenesis.”

**Table 1:** *FA-SAT* methylation analysis.

Sample		*FA-SAT*			Methylation %		Methylation variation of CpG Island
		DNA copy number	Long RNA	Small RNA	15 CpG sites	CpG Island	
1	DFT	=	-	+	52.2	62.8	=
	FMT				41.3	50.0	
2	DFT	=	=	=	71.5	79.2	-
	FMT				29.2	30.7	
3	DFT	-	+	-	74.6	74.3	=
	FMT				67.9	72.2	
6	DFT	-	+	=	67.9	70.9	-
	FMT				41.4	43.4	
13	DFT	=	-	-	71.4	75.6	-
	FMT				23.6	24.1	
14	DFT	-	+	+	69.8	77.2	-
	FMT				62.2	55.7	
15	DFT	=	=	+	50.0	56.4	=
	FMT				68.0	71.1	
16	DFT	=	-	=	57.5	64.6	=
	FMT				48.4	48.6	

*Presentation of each sample (DFT and FMT) with the FA-SAT DNA and RNA (long and small) status in the tumor samples (= maintenance, - decrease and + increase) and the percent values of methylation (CpG Total and Island) and the methylation status of each FMT sample, when compared with the respective DFT.*

## Discussion

The modulation of satDNA transcription by epigenetic mechanisms, which include DNA methylation modifications, is supported by experimental and empirical evidences (Bouzinba-Segard et al., 2006; Eymery et al., 2009b; Vourc’h and Biamonti, 2011; Ferreira et al., 2015). *FA-SAT* was defined as a GC-rich satDNA (Fanning, 1987; Pontius and O’Brien, 2009) exhibiting a CpG island, which suggests that DNA methylation may be responsible for its regulation (Chaves et al., 2017). With this premise in mind, we designed an experimental approach that encompassed different cellular models (Ferreira et al., 2013), with different applications (cf. Introduction), aiming to trace, for the first time, both *FA-SAT* DNA and RNA profiles and verify if this satDNA is in fact regulated by DNA methylation. Thus and as satDNA sequences are commonly amplified (Santos et al., 2006; Bersani et al., 2015), hypomethylated (Narayan et al., 1998; Jackson et al., 2004; Ehrlich, 2009; Tilman et al., 2012; Walton et al., 2014; Ferreira et al., 2015; Saksouk et al., 2015) and overexpressed in tumor cells (Ting et al., 2011; Tilman et al., 2012; Kondratova et al., 2014; Bersani et al., 2015; Zhu et al., 2018), we targeted these parameters regarding *FA-SAT* in: FCA primary cells (non-tumor); different passages of a feline mammary cell line-FkMTp; and several feline mammary spontaneous tumors (FMTs) using the respective disease-free tissues (DFTs) samples extracted from the same individuals but from a healthy mammary gland (providing an accurate and more realistic scenario).

Interestingly, and in contrast to the *FA-SAT* DNA amplification previously reported in a cat fibrosarcoma (Santos et al., 2006), only the latest passages (p70 and p112) of the FkMTp cell line showed an increase in *FA-SAT* DNA copy number. Regarding the transcription of this satDNA sequence, *FA-SAT* long (≥200 bp) and small (<200 bp) ncRNAs were found. Different authors also reported the existence/formation of satellite transcripts of different sizes (Bouzinba-Segard et al., 2006; Lu and Gilbert, 2007). *FA-SAT* small transcripts can be the result of: (1) a rapid turnover of *FA-SAT* long ncRNA, already described for centromeric transcripts (Choi et al., 2011; Chan et al., 2012; Rosic et al., 2014); (2) the processing of *FA-SAT* long ncRNA, which can also display a function, as reported for other satellite RNAs (Bouzinba-Segard et al., 2006; Lu and Gilbert, 2007). Functional studies will be essential to disclose and characterize the *FA-SAT* ncRNAs function(s) in tumor and non-tumor cells. When compared with the cat primary cells, the initial FkMTp passage 7, presented a decrease of *FA-SAT* long ncRNAs and a notorious increase of *FA-SAT* small ncRNAs. Moreover, FkMTp p21 presented an increase of both *FA-SAT* long and small ncRNAs, but the latest passages of FkMTp exhibited *FA-SAT* ncRNA levels closer to those of the cat primary non-tumor cells. Regarding the FMT samples, while some of the samples exhibited an increased level of either *FA-SAT* small or long RNAs, others maintained or even decreased the transcription of *FA-SAT*. When these *FA-SAT* expression levels were correlated with the variation in DNA copy number it does not seem to exist any association. These data are in fact against what was reported by Bersani et al. (2015) for human *SATII*. Thus, other mechanisms must be involved in the regulation of the *FA-SAT* transcription, such as epigenetic modifications, as already described for other satDNA sequences (Bouzinba-Segard et al., 2006; Alexiadis et al., 2007; Vourc’h and Biamonti, 2011; Cooper et al., 2014; Hall et al., 2017).

DNA methylation was previously suggested as a mechanism capable of regulating the transcription of satDNA sequences (Bouzinba-Segard et al., 2006; Alexiadis et al., 2007; Vourc’h and Biamonti, 2011). Additionally, the *FA-SAT* has in its monomer unit a CpG island (Chaves et al., 2017), making this satDNA a good candidate for this form of regulation. In our study, the methylation status of *FA-SAT* was thus estimated in both cellular models, the tumor and non-tumor cell lines and the spontaneous tumor and respective DFTs. The use of these cellular models provided a complementary approach. Specifically, in the tumor cell line, the cancer-driver mutations are responsible for the behavior of these cells that was possible to follow over time by the use of different passages. In addition, this *in vitro* system allowed to accomplish experiments impossible to perform in spontaneous tumor tissues, such as the global demethylation experiment. Complementarily, spontaneous tissue tumors also present acquired cancer-driver mutations, but also retain the cells of the tumor microenvironment that may influence the methylation status of the DNA sequences (Ting et al., 2011), which is actually lost in the *in vitro* cell culture.

Through the bisulfite sequencing analysis, it was possible to observe that the *FA-SAT* DNA sequence is methylated in the primary non-tumor cells and in the tumor cell line, with the FkMTp p21 being the one that presented the lowest sequence methylation level. Indeed, this cell line passage is the only one exhibiting increased levels of *FA-SAT* long ncRNAs, indicating that DNA methylation can be, in fact, an epigenetic regulator of *FA-SAT* transcription. In the FMTs, although *FA-SAT* DNA hypomethylation was observed in half of the samples analyzed, the methylation status did not seem to be the unique factor responsible for its expression once it was not possible to find a clear correlation between the methylation status and the *FA-SAT* expression levels. This is a different profile to what was observed for several others satDNA sequences in cancer, which were found hypomethylated (Narayan et al., 1998; Qu G. et al., 1999; Qu G.Z. et al., 1999; Saito et al., 2001; Wong et al., 2001; Jackson et al., 2004; Widschwendter et al., 2004; Ehrlich, 2005; Tilman et al., 2012; Enukashvily and Ponomartsev, 2013; Walton et al., 2014) and showed a concomitant increased expression (Ehrlich, 2009; Ting et al., 2011; Walton et al., 2014). Nevertheless, some other authors have reported a poor correlation between DNA hypomethylation and satDNA transcription in cancer cells (Alexiadis et al., 2007; Tilman et al., 2012), similarly to what we found. Tilman et al. (2012) proved that the hypomethylation of human *SATII* in cancer does not regulates its transcription and that it is initiated by the heat shock pathway activation. Nevertheless, it is also important to highlight that all the tumor samples used in the present work (and even the samples analyzed by other authors) should exhibit different cancer-driver mutations, resulting in these different scenarios. In this aspect, it will be very important to extensively sequence all these samples in order to get a clear picture about the genetic and epigenetic background of these cancer genomes. In the near future, with technologies such as nanopore sequencing (which also enable an epigenetic analysis of the sequences) (Rand et al., 2017), this might be a reality, and we could actually associate the cancer genome with the DNA methylation of a certain sequence and its expression phenotypes. In an attempt to partially overcome this difficulty, we performed a demethylation experiment in different cancer genomes that were related by the same initial genetic and epigenetic background; that is, the different passages of the tumor cell line FkMTp. The demethylation of a sequence using a global demethylation agent (i.e., Azacytidine) should unleash its transcription if the DNA methylation is its regulatory mechanism and/or if it is not dysregulated. Ting et al. (2011) used the same agent to prove that DNA methylation is the potential mechanism for the *in vitro* satDNA silencing and that its demethylation is responsible for the aberrant satellite overexpression detected in a variety of epithelial cancers (Ting et al., 2011). Other authors have also used this approach to demonstrate the expression modulation of others satDNAs’ by DNA methylation (Bouzinba-Segard et al., 2006; Eymery et al., 2009b). Thus, the demethylation of the *FA-SAT* DNA in the cell lines resulted in different scenarios: (1) in primary cells, *FA-SAT* transcription was derepressed, originating the increase of both small and long satncRNAs levels; (2) in the FkMTp tumor cells the *FA-SAT* overexpression is only observed regarding the small *FA-SAT* transcripts; (3) in FkMTp p21 cells, *FA-SAT* long transcripts’ levels decreased and the small transcripts highly increased.

Assembling all data we can suggest that in normal genomes the *FA-SAT* expression is modulated by DNA methylation events resulting in the accumulation of its transcripts, similarly to what occur in other satDNAs (Bouzinba-Segard et al., 2006; Eymery et al., 2009b). However, in tumor genomes (cell lines and tissues) the DNA methylation must be dysregulated by the cancer-driver mutations acquired and/or other regulation mechanisms should be considered in this type of cells. Though, it is important to note that these mutations didn’t cause a pervasive transcription of this satDNA and thus, this sequence is still modulated in its transcription, which suggests a putative function of these transcripts in the cells. In addition, the different situations observed with the *FA-SAT* methylation and expression experiments in the cancer genomes certainly reflect different mutation panels acquired by these different genomes and/or different tumor environments (in this case only for the FMT samples). Further, at this point it becomes important to highlight this last aspect, which can also explain the apparent discrepancy in the data acquired between the tumor cell line and the spontaneous tumor tissues. In the tumor tissues, the contribution of the tumor microenvironment has to be considered for the epigenetic modifications observed, and thus the data acquired refers to a mixed population of cells from the tumor itself and cells from the tumor microenvironment. In tumor cell lines, the data only result from the tumor cells in culture. Based on these considerations, we can affirm that, in fact, we are analyzing two very different situations and thus, it was not expected to acquire similar data.

The studies about the regulation of these sequences and its (dys)regulation in cancer are scarce, and this is due to the difficulty concerning its study and to the fact that they can present different regulatory mechanisms (Ferreira et al., 2015). As these sequences are located mainly at heterochromatic regions, their epigenetic regulation by modifications of histones and Polycomb proteins was also suggested (Pruitt et al., 2006; Frescas et al., 2008; Eymery et al., 2009a; Almouzni and Probst, 2011; Bulut-Karslioglu et al., 2012; Hall et al., 2017). Thus, future work will focus on the discovery of alternative mechanisms that could explain the transcriptional regulation of *FA-SAT* DNA in cat cancer cells, since this organism is a promising animal model for the process of tumorigenesis due to its high genetic similarities with the human counterpart.

## Conclusion

In summary, the quantification of the *FA-SAT* DNA and RNAs levels and the *FA-SAT* methylation status analysis revealed that: (1) the number of *FA-SAT* DNA monomer copies is not related with the *FA-SAT* overexpression; (2) in most of the tumor samples analyzed the *FA-SAT* sequences are not hypomethylated; (3) in normal genomes the expression of *FA-SAT* seems to be modulated by DNA methylation; (4) in tumor cells the *FA-SAT* is still modulated in its expression since there is not a pervasive transcription of this satDNA; (5) however, in these tumor samples the transcription of *FA-SAT* does not seems to correlate well with the methylation status of the sequence, and so, other mechanisms should be considered. Finally, our work also highlights the importance of using different cellular models in this type of studies, since they complement each other, allowing to analyze different situations of the tumorigenesis process.

## Ethics Statement

This study was carried out in accordance with the animal research recommendations (EU Directive 2010/63/EU) and the samples and cell lines were provided in the frame of the research projects from the Science and Technology Foundation (FCT) from Portugal (Grant No. POCI/CVT/62940/04) and approved by the “Universidade de Trás-os-Montes e Alto Douro”.

## Author Contributions

RC conceptualized the work. DF, AE, FA, and RC provided the methodology. DF, AE, and RC contributed to validation. DF performed the formal analysis. DF, AE, and RC contributed to the investigation process. RC gathered resources. DF prepared and wrote and the original draft. AE, FA, and RC wrote, reviewed, and edited the manuscript. FA and RC supervised the work. RC administrated the project. RC acquired funding.

## Conflict of Interest Statement

The authors declare that the research was conducted in the absence of any commercial or financial relationships that could be construed as a potential conflict of interest.
