# Supplementary material for: DNA Methylation Patterns of a Satellite Non-coding Sequence – FA-SAT in Cancer Cells: Its Expression Cannot Be Explained Solely by DNA Methylation
Source: Front Genet. 2019 Feb 12;10:101. doi: 10.3389/fgene.2019.00101 (PMC6379292; doi:10.3389/fgene.2019.00101)
Supplement: Supplementary file 1 [file Data_Sheet_1.docx]

Supplementary Material

DNA methylation patterns of a satellite non-coding sequence - *FA-SAT* in cancer cells: its expression cannot be explained solely by DNA methylation

Daniela Ferreira, Ana Escudeiro, Filomena Adega, Raquel Chaves*

*** Correspondence:** Raquel Chaves: rchaves@utad.pt

# Supplementary Figures and Tables

## Supplementary Figures


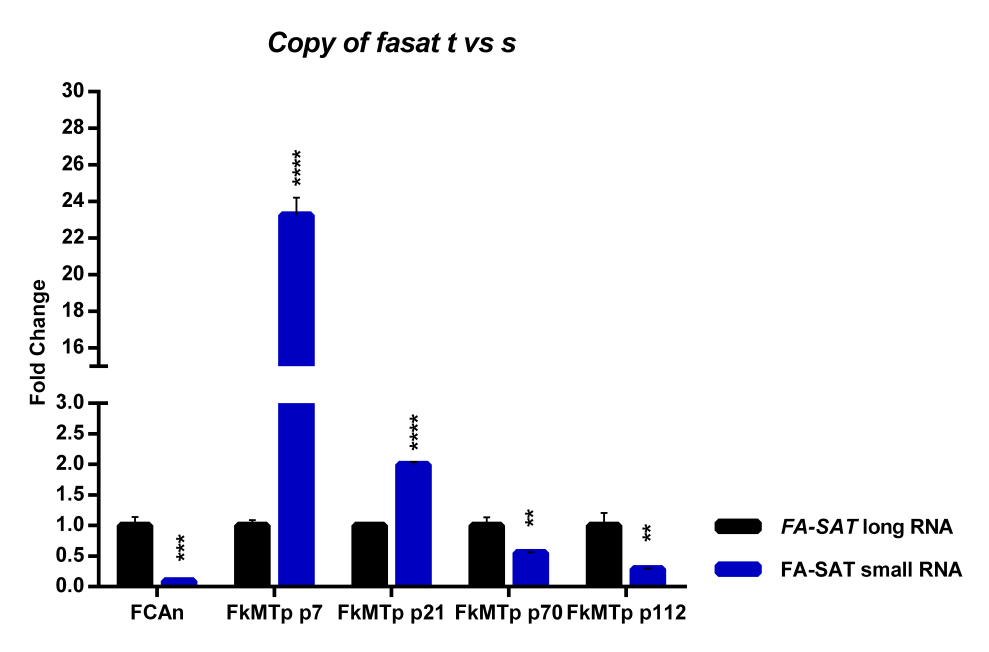


**Supplementary Figure 1.** *FA-SAT* long and small RNA. Relative quantification of *FA-SAT* long and small RNA in FCAn and in the different passages of FkMTp, using the *FA-SAT* long RNA as the reference. Data are presented as the means ± s.d. of three replicates. **p ≤ 0.01 ***p ≤ 0.001 ****p ≤ 0.0001 as determined by the student’s T-test.


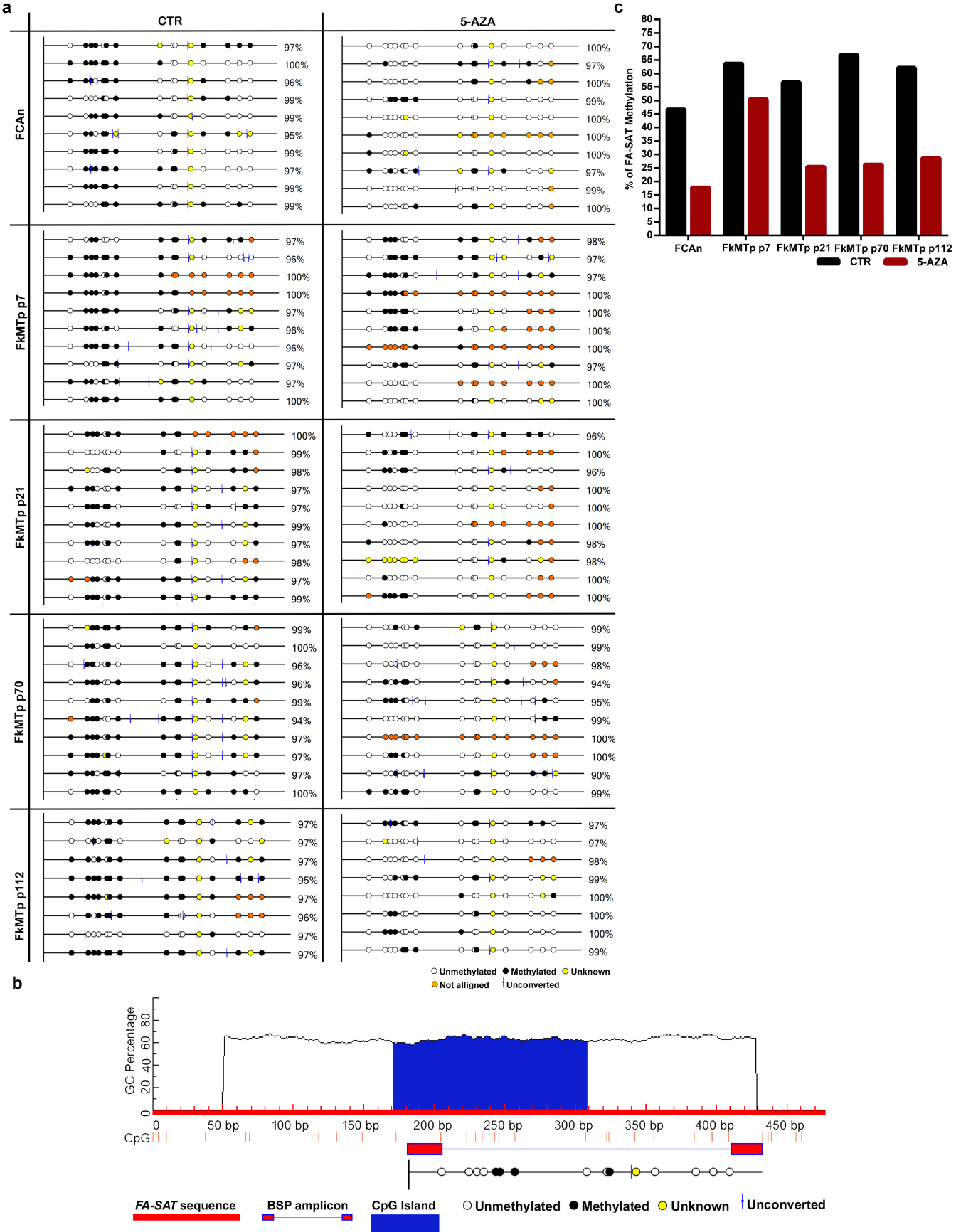


**Supplementary Figure 2.** Analysis of *FA-SAT* DNA methylation in the primary and FkMTp cells. (a) *FA-SAT* methylation status analysis by bisulfite sequencing in FCAn and p7, p21, p70 and p112 of FkMTp cell line in the control (CTR) and 5-AZA. These data were obtained using the MethylViewer software ([Pardo et al., 2011](#_ENREF_2)). The analysis of these data is represented in Fig. 2 a-b and in this Supplementary Fig. 1b. (b) Schematic representation of the CpG analysis showing the CpG profile (CpG sites as red bars) of the *FA-SAT* monomer in red (GenBank, sequence accession number: X06372.1), the CpG island in blue BSP primers (supplementary table 1) location. Also, it it represented an example of the MethylViewer software ([Pardo et al., 2011](#_ENREF_2)) output. The scheme presented was adapted from the output of MethPrimer ([Li and Dahiya, 2002](#_ENREF_1)) for CpG island prediction. (c) Graphical representation of the *FA-SAT* methylation percent values of the total analyzed CpG sequence observed by bisulfite sequencing of FCAn and the different passages of FkMTp in the control and 5-AZA.


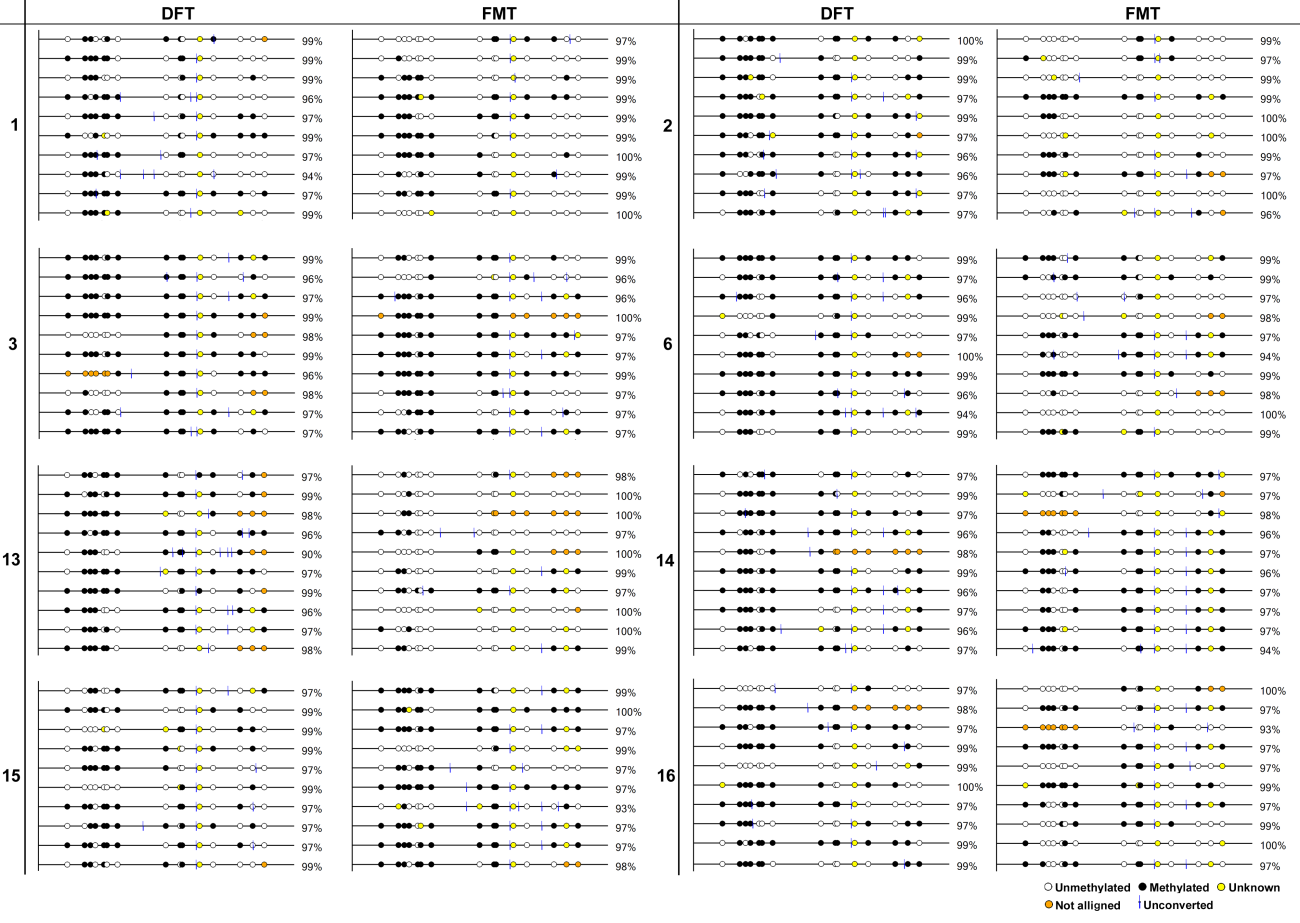


**Supplementary Figure 3.** Analysis of *FA-SAT* DNA methylation status by bisulfite sequencing of the FMT and DFT samples from the same individual (n=8). These data were obtained using the MethylViewer software ([Pardo et al., 2011](#_ENREF_2)). The analysis of these data is represented in Figure 4.

## Supplementary Tables

**Supplementary Table 1.** Sequence of the primers used in this work.

|  | Forward | Reverse |
| --- | --- | --- |
| *FA-SAT* BSP | TGGTATGGGTTTTTATTTTAGTTTT | AAAAAACCTCTACAATCCTCTCC |
| *FA-SAT*_83bpB | AGCTAAGGCTCTCCCCTCATG | TCAGCCTGCACCGCTTCT |

**Supplementary Table 2.** Standard curve parameters.

|  | R^2^ | Efficiency (%) |
| --- | --- | --- |
| *FA-SAT DNA* | 0.99 | 95.50 |
| *FA-SAT RNA* | 0.99 | 98.23 |

**Supplementary Table 3.** Relative quantification of *FA-SAT* Copy number, *FA-SAT* long and small RNA of the FkMTp passages by real-time RT-qPCR considering the cat primary cells (FCAn) genome as a reference. Values are mean ± SD.

|  | *FA-SAT* | | |
| --- | --- | --- | --- |
|  | Copy Number | Long RNA | Small RNA |
| FCAn | 1.00 (±0.13) | 1.00 (±0.14) | 1.00 (±9.87x10^-4^) |
| FkMTp p7 | 1.03 (±0.03) | 0.22 (±0.02) | 55.72 (±2.29) |
| FkMTp p21 | 1.42 (±0.03) | 2.00 (±2.11x10^-6^) | 42.82 (±1.03) |
| FkMTp p70 | 4.00 (±0.30) | 0.64 (±0.09) | 3.89 (±4.31x10^-3^) |
| FkMTp p112 | 3.10 (±0.49) | 0.56 (±0.12) | 1.77 (±0.03) |

**Supplementary Table 4.** Relative quantification of *FA-SAT* long and small RNA of FCAn and FkMTp passages in the CTR (control) and AZA conditions by real-time RT-qPCR considering the CTR as a reference. Values are mean ± SD.

|  |  | *FA-SAT* | |
| --- | --- | --- | --- |
|  |  | Long RNA | Small RNA |
| FCAn | CTR | 1.00 (±0.10) | 1.00 (±0.10) |
|  | AZA | 8.99 (±0.55) | 4.54 (±0.25) |
| FkMTp p7 | CTR | 1.00 (±0.09) | 1.00 (±0.04) |
|  | AZA | 1.10 (±0.09) | 7.11 (±0.18) |
| FkMTp p21 | CTR | 1.00 (±1.18x10^-6^) | 1.00 (±0.02) |
|  | AZA | 0.09 (±6.10x10^-3^) | 21.65 (±0.22) |
| FkMTp p70 | CTR | 1.00 (±0.18) | 1.00 (±0.01) |
|  | AZA | 0.93 (±0.03) | 9.66 (±0.43) |
| FkMTp p112 | CTR | 1.00 (±0.18) | 1.00 (±0.02) |
|  | AZA | 0.58 (±0.03) | 15.01 (±0.62) |

**Supplementary Table 5.** *FA-SAT* DNA and RNAs (long and small) quantification of each FMT sample using the DFT sample from the same individual as a reference. Values are mean ± SD.

|  | *FA-SAT* DNA copy number | | *FA-SAT* long RNA | | *FA-SAT* small RNA | |
| --- | --- | --- | --- | --- | --- | --- |
|  | DFT | FMT | DFT | FMT | DFT | FMT |
| 1 | 1.00 (±0.12) | 1.21 (±0.04) | 1.00 (±0.06) | 0.04 (±0.00) | 1.00 (±0.02) | 104.32 (±3.44) |
| 2 | 1.00 (±0.17) | 0.84 (±0.04) | 1.00 (±0.10) | 1.30 (±0.35) | 1.00 (±0.05) | 1.01 (±0.02) |
| 3 | 1.00 (±0.06) | 0.43 (±0.04) | 1.00 (±0.10) | 7.44 (±0.54) | 1.00 (±0.03) | 0.06 (±5.77x10^-3^) |
| 6 | 1.00 (±0.03) | 0.48 (±0.02) | 1.00 (±0.09) | 13.24 (±0.68) | 1.00 (±0.02) | 1.53 (±0.08) |
| 13 | 1.00 (±0.07) | 0.71 (±0.07) | 1.00 (±0.04) | 0.39 (±0.03) | 1.00 (±0.03) | 0.05 (±0.00)0 |
| 14 | 1.00 (±0.11) | 0.38 (±0.06) | 1.00 (±0.09) | 92.29 (±3.61) | 1.00 (±0.09) | 5.82 (±0.18) |
| 15 | 1.00 (±0.11) | 1.25 (±0.06) | 1.00 (±0.03) | 1.14 (±0.17) | 1.00 (±0.16) | 7.64 (±0.16) |
| 16 | 1.00 (±0.16) | 0.73 (±0.07) | 1.00 (±0.03) | 0.20 (±0.04) | 1.00 (±0.02) | 1.05 (±0.06) |

# Refererences

Li, L.C., and Dahiya, R. (2002). MethPrimer: designing primers for methylation PCRs. *Bioinformatics* 18(11)**,** 1427-1431.

Pardo, C.E., Carr, I.M., Hoffman, C.J., Darst, R.P., Markham, A.F., Bonthron, D.T., et al. (2011). MethylViewer: computational analysis and editing for bisulfite sequencing and methyltransferase accessibility protocol for individual templates (MAPit) projects. *Nucleic Acids Res* 39(1)**,** e5. doi: 10.1093/nar/gkq716.
